# Data on some qualitative parameters of Carolea olive oils obtained in different areas of Calabria (Southern Italy)

**DOI:** 10.1016/j.dib.2016.08.009

**Published:** 2016-08-09

**Authors:** Amalia Piscopo, Alessandra De Bruno, Angela Zappia, Carmine Ventre, Marco Poiana

**Affiliations:** a Department of AGRARIA, University Mediterranea of Reggio Calabria, 89124 Vito, Reggio Calabria, Italy; b Centro Analisi Biochimiche Sas, Via Pitagora 4, 89016 Rizziconi, Reggio Calabria, Italy

## Abstract

This data article contains complementary results related to the paper “Characterization of monovarietal olive oils obtained from mills of Calabria region (Southern Italy)” (Piscopo et al., 2016) [1]. Data was obtained by capillary-column gas chromatography, analyzing sterols and triterpene dialcohols and ethyl esters in the composition of Carolea olive oils. They were produced in different areas of Calabria region (Southern Italy), named: the Sibari׳s plateau (SP), the Valley of Sant׳ Eufemia (VSE), the Tyrrhenian southern area (TSA), the Ionian southern coast (ISC) and the Ionian area of Catanzaro (IAC). Specifically the characterized samples were 24 in the SP; 43 in the VSE; 15 in the TSA; 30 in the ISC, and 34 in the IAC, for a total amount of 146 olive oils. The differences in some compositional characteristics denoted the effect of the environmental and could be considered to improve the local productions. The compilation of this data provides a resource for the wider research community and the interpretation of this data could be found in the research article noted above.

**Specifications Table**

**Table:** 

Subject area	*Chemistry*
More specific subject area	*Composition*
Type of data	*Table*
How data was acquired	*Gas Chromatography (GC thermo OnColumn-FID)*
Data format	*Raw, analyzed*
Experimental factors	*Oil samples (cv Carolea) were sampled in different farms of the Calabria region and directly submitted to GC analysis*
Experimental features	*Analyses of olive oils and comparison among growing area of production*
Data source location	*Reggio Calabria, Catanzaro, Cosenza, Crotone, Vibo Valentia, Italy*
Data accessibility	*Data is with this article*

**Value of the data**

• The data provides some additional data on composition of Carolea oils in Calabria.
• The data denoted different olive growing practices and processing in this region of the Southern Italy, manifested for ethyl ester quantification by the showed high standard deviations.
• This data could serve as a benchmark for other researchers, evidencing the peculiar characteristics of this olive cultivar and the environmental influences.

## 1 Data

Supplementary material S1 reports the composition of sterols in the olive oils cv Carolea sampled in different growing areas of Calabria (Southern Italy).

Supplementary material S2 reports the triterpene dialcohols in the olive oils cv Carolea sampled in different growing areas of Calabria (Southern Italy).

Supplementary material S3 reports the ethyl esters in the olive oils cv Carolea sampled in different growing areas of Calabria (Southern Italy).

Table 1 reports the data expressed as mean and standard deviation of the sterols, triterpene dialcohols and ethyl esters in Carolea oils.

## 2 Experimental design, materials and methods

The sampling of olive oils in different areas of Calabria region (Italy) was conducted as illustrated by Piscopo et al. [1]. The analysis of sterols, triterpene dialcohols and ethyl esters in olive oil samples were performed according to European Regulations [2]. One-way analysis of variance (ANOVA) and post-hoc Duncan test (significant level for *P*<0.05) were applied to the data by using of SPSS Software (Version 15.0, SPSS Inc., Chicago, IL, USA). Different letters indicate significant differences among samples.

## Figures and Tables

**Table 1:** Data on sterols, triterpene dialcohols and ethyl esters of monovarietal olive oils produced in Calabria (Southern Italy). SP (Sibari׳s Plateau). VSE (Valley of Sant׳Eufemia). TSA (Tyrrhenian Southern area). ISC (Ionian Southern coast). IAC (Ionian Area of Catanzaro).

Growing area	SP		VSE		TSA		ISC		IAC		Sign
	M	D.S.	M	D.S.	M	D.S.	M	D.S.	M	D.S.	
Cholesterol	0,09	0,02b	0,08	0,01b	0,09	0,01a	0,09	0,01a	0,09	0,01b	**
Brassicasterol	0,00	0,00b	0,00	0,00b	0,01	0,01a	0,00	0,00b	0,00	0,00b	**
2,4 Methylencolesterol	0,11	0,01b	0,11	0,01b	0,12	0,02a	0,11	0,01b	0,11	0,01b	*
Campesterol	2,35	0,32b	2,29	0,47b	1,91	0,51c	2,64	0,48a	2,31	0,47b	**
Campestanol	0,15	0,01	0,15	0,01	0,15	0,03	0,15	0,01	0,15	0,01	n.s
Stigmasterol	0,90	0,26b	0,82	0,27b	0,85	0,22b	1,10	0,23a	0,77	0,19b	**
Clerosterol	0,88	0,10b	0,88	0,07b	0,96	0,14a	0,86	0,05b	0,86	0,05b	**
Betasitosterol	86,97	2,28ab	87,36	1,18a	87,01	1,98ab	86,18	2,72b	87,46	0,89a	*
Sitostanol	1,04	0,17	1,00	0,17	1,13	0,23	0,99	0,21	1,00	0,12	n.s.
D 5 Avenasterol	6,02	2,28	5,86	1,16	6,22	1,16	6,35	2,90	5,81	0,92	n.s.
D 5,24 Stigmastadienol	0,87	0,09b	0,84	0,06bc	0,92	0,15a	0,86	0,05bc	0,82	0,07c	**
D 7 Stigmastenol	0,18	0,05ab	0,16	0,04b	0,15	0,04b	0,20	0,06a	0,17	0,04b	*
D 7 Avenasterol	0,44	0,05	0,44	0,04	0,47	0,07	0,46	0,11	0,46	0,07	n.s.
Eritrodiol + Uvaol	2,42	0,41b	2,23	0,31b	2,73	0,82a	2,47	0,40b	2,30	0,38b	**
Eritrodiol + Uvaol (mg/kg)	44,19	9,15	40,14	9,05	46,53	25,79	45,56	9,18	40,81	6,67	n.s.
Ethyl Palmitate	2,45	1,70b	3,09	3,99b	0,99	0,81b	5,16	4,33a	2,74	3,82b	**
Ethyl Oleate	6,47	5,91	6,01	9,78	1,50	1,94	8,90	10,49	6,87	10,87	n.s.

** Significance at P < 0.01; * Significance at P < 0.05; n.s. not significant. Results followed by different letters are significantly different by Duncan post-hoc test.
