# Supplementary material for: Data on some qualitative parameters of Carolea olive oils obtained in different areas of Calabria (Southern Italy)
Source: Data Brief. 2016 Aug 9;9:78–80. doi: 10.1016/j.dib.2016.08.009 (PMC5021706; doi:10.1016/j.dib.2016.08.009)
Supplement: Supplementary file 2 — Supplementary material [file mmc2.doc]

| Samples | Cholesterol | Brassicasterol | 2,4 Methylene cholesterol | Campesterol | Campestanol | Stigmasterol | Clerosterol | Betasitosterol | Sitostanol | D 5 Avenasterol | D 5,24 Stigmastadienol | D 7 Stigmastenol | D 7 Avenasterol |
| --- | --- | --- | --- | --- | --- | --- | --- | --- | --- | --- | --- | --- | --- |
| SP-1 | 0,11 | 0,01 | 0,13 | 2,16 | 0,13 | 1,13 | 1,05 | 85,70 | 1,31 | 6,56 | 1,05 | 0,11 | 0,52 |
| SP-2 | 0,11 | 0,00 | 0,13 | 2,21 | 0,13 | 1,64 | 1,05 | 85,12 | 1,31 | 6,57 | 1,05 | 0,13 | 0,53 |
| SP-3 | 0,13 | 0,01 | 0,13 | 2,55 | 0,13 | 1,46 | 1,05 | 85,01 | 1,31 | 6,56 | 1,05 | 0,08 | 0,52 |
| SP-4 | 0,12 | 0,01 | 0,13 | 1,99 | 0,13 | 1,17 | 1,05 | 85,79 | 1,31 | 6,56 | 1,05 | 0,18 | 0,52 |
| SP-5 | 0,09 | 0,00 | 0,10 | 2,91 | 0,15 | 1,04 | 0,81 | 87,43 | 1,01 | 5,05 | 0,81 | 0,19 | 0,40 |
| SP-6 | 0,08 | 0,00 | 0,10 | 2,06 | 0,15 | 0,56 | 0,82 | 88,68 | 1,03 | 5,14 | 0,82 | 0,13 | 0,41 |
| SP-7 | 0,08 | 0,00 | 0,11 | 2,33 | 0,17 | 0,58 | 0,89 | 87,75 | 1,11 | 5,55 | 0,89 | 0,11 | 0,44 |
| SP-8 | 0,09 | 0,00 | 0,10 | 2,98 | 0,15 | 0,78 | 0,82 | 87,55 | 1,02 | 5,10 | 0,82 | 0,18 | 0,41 |
| SP-9 | 0,08 | 0,00 | 0,10 | 2,09 | 0,15 | 0,70 | 0,81 | 88,61 | 1,01 | 5,06 | 0,81 | 0,17 | 0,40 |
| SP-10 | 0,07 | 0,00 | 0,10 | 2,19 | 0,15 | 0,84 | 0,82 | 88,14 | 1,03 | 5,15 | 0,82 | 0,25 | 0,41 |
| SP-11 | 0,07 | 0,00 | 0,10 | 2,09 | 0,16 | 0,74 | 0,83 | 88,34 | 1,04 | 5,20 | 0,83 | 0,18 | 0,42 |
| SP-12 | 0,08 | 0,00 | 0,10 | 2,66 | 0,15 | 0,76 | 0,82 | 87,81 | 1,03 | 5,13 | 0,82 | 0,23 | 0,41 |
| SP-13 | 0,07 | 0,00 | 0,10 | 2,77 | 0,15 | 1,10 | 0,80 | 87,67 | 1,00 | 4,99 | 0,80 | 0,16 | 0,40 |
| SP-14 | 0,06 | 0,00 | 0,13 | 1,73 | 0,14 | 0,81 | 1,05 | 78,48 | 0,49 | 15,36 | 1,00 | 0,19 | 0,56 |
| SP-15 | 0,06 | 0,00 | 0,11 | 2,84 | 0,16 | 0,76 | 0,86 | 87,18 | 1,07 | 5,37 | 0,86 | 0,30 | 0,43 |
| SP-16 | 0,11 | 0,00 | 0,10 | 1,99 | 0,15 | 0,81 | 0,78 | 88,93 | 0,98 | 4,89 | 0,78 | 0,10 | 0,39 |
| SP-17 | 0,09 | 0,00 | 0,10 | 2,42 | 0,16 | 1,06 | 0,83 | 87,64 | 1,04 | 5,18 | 0,83 | 0,24 | 0,41 |
| SP-18 | 0,07 | 0,00 | 0,11 | 2,56 | 0,16 | 0,81 | 0,84 | 87,65 | 1,05 | 5,27 | 0,84 | 0,21 | 0,42 |
| SP-19 | 0,09 | 0,00 | 0,10 | 2,40 | 0,15 | 0,89 | 0,81 | 88,08 | 1,01 | 5,06 | 0,81 | 0,19 | 0,40 |
| SP-20 | 0,09 | 0,00 | 0,10 | 2,40 | 0,15 | 0,79 | 0,82 | 88,11 | 1,02 | 5,12 | 0,82 | 0,18 | 0,41 |
| SP-21 | 0,07 | 0,00 | 0,11 | 2,20 | 0,15 | 0,79 | 0,93 | 83,22 | 0,76 | 10,16 | 0,91 | 0,21 | 0,49 |
| SP-22 | 0,07 | 0,00 | 0,10 | 2,14 | 0,16 | 0,79 | 0,83 | 88,24 | 1,04 | 5,18 | 0,83 | 0,21 | 0,41 |
| SP-23 | 0,08 | 0,00 | 0,10 | 2,53 | 0,15 | 0,74 | 0,81 | 88,08 | 1,02 | 5,08 | 0,81 | 0,18 | 0,41 |
| SP-24 | 0,08 | 0,00 | 0,10 | 2,18 | 0,16 | 0,79 | 0,84 | 88,15 | 1,05 | 5,24 | 0,84 | 0,16 | 0,42 |
| VSE-1 | 0,10 | 0,01 | 0,13 | 2,29 | 0,13 | 0,97 | 1,05 | 85,64 | 1,31 | 6,57 | 1,05 | 0,22 | 0,53 |
| VSE-2 | 0,09 | 0,02 | 0,13 | 1,91 | 0,13 | 1,02 | 1,05 | 86,00 | 1,31 | 6,57 | 1,05 | 0,19 | 0,53 |
| VSE-3 | 0,08 | 0,00 | 0,10 | 2,20 | 0,15 | 0,74 | 0,80 | 88,54 | 1,01 | 5,03 | 0,80 | 0,14 | 0,40 |
| VSE-4 | 0,07 | 0,00 | 0,10 | 2,69 | 0,16 | 0,84 | 0,83 | 87,71 | 1,04 | 5,20 | 0,83 | 0,10 | 0,42 |
| VSE-5 | 0,10 | 0,00 | 0,11 | 1,83 | 0,14 | 2,05 | 1,00 | 82,88 | 1,08 | 9,41 | 0,76 | 0,18 | 0,46 |
| VSE-6 | 0,10 | 0,00 | 0,13 | 2,03 | 0,15 | 0,70 | 1,08 | 84,14 | 0,65 | 9,40 | 0,83 | 0,19 | 0,60 |
| VSE-7 | 0,10 | 0,00 | 0,06 | 2,14 | 0,13 | 1,17 | 1,05 | 85,06 | 0,47 | 8,35 | 0,84 | 0,19 | 0,45 |
| VSE-8 | 0,09 | 0,00 | 0,10 | 1,54 | 0,16 | 1,36 | 0,83 | 88,35 | 1,04 | 5,18 | 0,83 | 0,10 | 0,41 |
| VSE-9 | 0,08 | 0,00 | 0,10 | 2,70 | 0,15 | 0,66 | 0,81 | 87,97 | 1,02 | 5,08 | 0,81 | 0,21 | 0,41 |
| VSE-10 | 0,09 | 0,00 | 0,10 | 3,04 | 0,15 | 0,58 | 0,81 | 87,74 | 1,01 | 5,05 | 0,81 | 0,21 | 0,40 |
| VSE-11 | 0,08 | 0,00 | 0,11 | 2,34 | 0,16 | 0,57 | 0,85 | 88,10 | 1,06 | 5,29 | 0,85 | 0,18 | 0,42 |
| VSE-12 | 0,09 | 0,00 | 0,10 | 3,00 | 0,15 | 0,77 | 0,82 | 87,49 | 1,03 | 5,13 | 0,82 | 0,18 | 0,41 |
| VSE-13 | 0,10 | 0,01 | 0,10 | 3,20 | 0,15 | 0,39 | 0,80 | 87,76 | 1,00 | 5,00 | 0,80 | 0,29 | 0,40 |
| VSE-14 | 0,06 | 0,00 | 0,11 | 2,74 | 0,16 | 0,50 | 0,86 | 87,74 | 1,07 | 5,35 | 0,86 | 0,13 | 0,43 |
| VSE-15 | 0,08 | 0,00 | 0,11 | 1,82 | 0,17 | 0,79 | 0,88 | 88,04 | 1,11 | 5,53 | 0,88 | 0,14 | 0,44 |
| VSE-16 | 0,08 | 0,00 | 0,11 | 1,73 | 0,16 | 0,89 | 0,87 | 88,24 | 1,08 | 5,41 | 0,87 | 0,13 | 0,43 |
| VSE-17 | 0,09 | 0,00 | 0,10 | 2,99 | 0,15 | 0,67 | 0,82 | 87,54 | 1,03 | 5,14 | 0,82 | 0,23 | 0,41 |
| VSE-18 | 0,07 | 0,00 | 0,11 | 1,74 | 0,12 | 0,95 | 0,86 | 85,71 | 0,56 | 8,52 | 0,74 | 0,17 | 0,46 |
| VSE-19 | 0,08 | 0,00 | 0,11 | 2,72 | 0,16 | 0,71 | 0,84 | 87,57 | 1,06 | 5,28 | 0,84 | 0,21 | 0,42 |
| VSE-20 | 0,08 | 0,00 | 0,11 | 2,40 | 0,17 | 0,67 | 0,90 | 87,42 | 1,13 | 5,65 | 0,90 | 0,11 | 0,45 |
| VSE-21 | 0,08 | 0,00 | 0,11 | 2,04 | 0,16 | 1,03 | 0,85 | 87,89 | 1,07 | 5,33 | 0,85 | 0,17 | 0,43 |
| VSE-22 | 0,08 | 0,00 | 0,11 | 2,22 | 0,16 | 0,76 | 0,86 | 87,89 | 1,08 | 5,39 | 0,86 | 0,16 | 0,43 |
| VSE-23 | 0,08 | 0,00 | 0,11 | 1,38 | 0,17 | 0,87 | 0,90 | 88,27 | 1,13 | 5,63 | 0,90 | 0,11 | 0,45 |
| VSE-24 | 0,09 | 0,00 | 0,10 | 1,65 | 0,16 | 0,86 | 0,83 | 88,71 | 1,04 | 5,21 | 0,83 | 0,10 | 0,42 |
| VSE-25 | 0,08 | 0,00 | 0,11 | 1,86 | 0,16 | 0,75 | 0,87 | 88,17 | 1,09 | 5,46 | 0,87 | 0,13 | 0,44 |
| VSE-26 | 0,09 | 0,00 | 0,11 | 2,12 | 0,15 | 0,72 | 0,94 | 86,41 | 0,83 | 7,15 | 0,82 | 0,16 | 0,50 |
| VSE-27 | 0,08 | 0,00 | 0,08 | 2,41 | 0,14 | 1,01 | 0,94 | 86,41 | 0,76 | 6,76 | 0,84 | 0,14 | 0,43 |
| VSE-28 | 0,08 | 0,00 | 0,10 | 2,13 | 0,15 | 1,01 | 0,82 | 88,17 | 1,03 | 5,13 | 0,82 | 0,14 | 0,41 |
| VSE-29 | 0,09 | 0,00 | 0,10 | 2,30 | 0,15 | 0,97 | 0,82 | 88,05 | 1,02 | 5,12 | 0,82 | 0,14 | 0,41 |
| VSE-30 | 0,08 | 0,00 | 0,10 | 2,52 | 0,16 | 0,62 | 0,83 | 88,03 | 1,04 | 5,18 | 0,83 | 0,19 | 0,41 |
| VSE-31 | 0,08 | 0,00 | 0,10 | 2,68 | 0,16 | 0,67 | 0,83 | 87,79 | 1,04 | 5,21 | 0,83 | 0,18 | 0,42 |
| VSE-32 | 0,08 | 0,01 | 0,10 | 2,98 | 0,16 | 0,44 | 0,83 | 87,75 | 1,03 | 5,17 | 0,83 | 0,21 | 0,41 |
| VSE-33 | 0,08 | 0,00 | 0,11 | 2,04 | 0,16 | 0,72 | 0,86 | 88,17 | 1,07 | 5,35 | 0,86 | 0,16 | 0,43 |
| VSE-34 | 0,08 | 0,00 | 0,11 | 2,62 | 0,16 | 0,91 | 0,87 | 87,28 | 1,09 | 5,43 | 0,87 | 0,15 | 0,43 |
| VSE-35 | 0,08 | 0,00 | 0,11 | 2,06 | 0,14 | 0,81 | 0,88 | 86,55 | 0,83 | 7,13 | 0,82 | 0,12 | 0,46 |
| VSE-36 | 0,08 | 0,00 | 0,10 | 1,69 | 0,14 | 0,91 | 0,85 | 87,22 | 0,80 | 6,85 | 0,79 | 0,13 | 0,44 |
| VSE-37 | 0,08 | 0,00 | 0,11 | 1,80 | 0,14 | 0,85 | 0,87 | 86,92 | 0,82 | 7,01 | 0,80 | 0,15 | 0,45 |
| VSE-38 | 0,08 | 0,00 | 0,11 | 2,57 | 0,16 | 0,69 | 0,87 | 87,52 | 1,09 | 5,46 | 0,87 | 0,14 | 0,44 |
| VSE-39 | 0,08 | 0,00 | 0,12 | 3,35 | 0,16 | 1,16 | 0,95 | 87,37 | 0,77 | 4,73 | 0,67 | 0,25 | 0,38 |
| VSE-40 | 0,08 | 0,00 | 0,11 | 2,30 | 0,16 | 0,73 | 0,86 | 87,87 | 1,07 | 5,36 | 0,86 | 0,17 | 0,43 |
| VSE-41 | 0,08 | 0,00 | 0,11 | 2,01 | 0,16 | 0,77 | 0,87 | 88,09 | 1,08 | 5,42 | 0,87 | 0,11 | 0,43 |
| VSE-42 | 0,08 | 0,00 | 0,11 | 2,13 | 0,17 | 0,71 | 0,89 | 87,82 | 1,11 | 5,55 | 0,89 | 0,10 | 0,44 |
| VSE-43 | 0,09 | 0,00 | 0,11 | 2,71 | 0,16 | 0,67 | 0,86 | 87,50 | 1,08 | 5,38 | 0,86 | 0,16 | 0,43 |
| VSE-44 | 0,07 | 0,00 | 0,11 | 2,03 | 0,16 | 0,64 | 0,84 | 88,47 | 1,05 | 5,26 | 0,84 | 0,10 | 0,42 |
| TSA-1 | 0,10 | 0,01 | 0,13 | 2,28 | 0,13 | 1,15 | 1,05 | 85,48 | 1,31 | 6,56 | 1,05 | 0,21 | 0,53 |
| TSA-2 | 0,11 | 0,01 | 0,13 | 1,96 | 0,13 | 1,13 | 1,05 | 85,85 | 1,31 | 6,57 | 1,05 | 0,17 | 0,53 |
| TSA-3 | 0,11 | 0,01 | 0,13 | 1,60 | 0,13 | 1,15 | 1,05 | 86,22 | 1,31 | 6,56 | 1,05 | 0,14 | 0,53 |
| TSA-4 | 0,11 | 0,01 | 0,13 | 1,73 | 0,13 | 1,08 | 1,05 | 86,13 | 1,31 | 6,57 | 1,05 | 0,17 | 0,53 |
| TSA-5 | 0,11 | 0,01 | 0,13 | 2,07 | 0,13 | 1,08 | 1,05 | 85,75 | 1,31 | 6,56 | 1,05 | 0,21 | 0,53 |
| TSA-6 | 0,10 | 0,00 | 0,07 | 2,32 | 0,15 | 0,56 | 1,02 | 85,78 | 0,77 | 7,88 | 0,73 | 0,19 | 0,42 |
| TSA-7 | 0,10 | 0,00 | 0,16 | 2,13 | 0,16 | 0,55 | 1,07 | 85,17 | 0,73 | 8,55 | 0,73 | 0,22 | 0,44 |
| TSA-8 | 0,07 | 0,02 | 0,10 | 1,05 | 0,14 | 0,57 | 0,76 | 90,36 | 0,95 | 4,76 | 0,76 | 0,10 | 0,38 |
| TSA-9 | 0,11 | 0,03 | 0,16 | 2,95 | 0,23 | 0,85 | 1,24 | 83,11 | 1,55 | 7,75 | 1,24 | 0,16 | 0,62 |
| TSA-10 | 0,09 | 0,00 | 0,10 | 1,43 | 0,15 | 0,70 | 0,82 | 89,23 | 1,02 | 5,12 | 0,82 | 0,10 | 0,41 |
| TSA-11 | 0,08 | 0,00 | 0,10 | 1,39 | 0,15 | 0,64 | 0,81 | 89,48 | 1,01 | 5,03 | 0,81 | 0,10 | 0,40 |
| TSA-12 | 0,09 | 0,00 | 0,11 | 1,48 | 0,17 | 0,79 | 0,89 | 88,40 | 1,11 | 5,54 | 0,89 | 0,11 | 0,44 |
| TSA-13 | 0,08 | 0,00 | 0,11 | 1,68 | 0,16 | 0,82 | 0,84 | 88,60 | 1,06 | 5,28 | 0,84 | 0,11 | 0,42 |
| TSA-14 | 0,10 | 0,00 | 0,10 | 2,65 | 0,16 | 0,86 | 0,84 | 87,60 | 1,05 | 5,24 | 0,84 | 0,14 | 0,42 |
| TSA-15 | 0,09 | 0,00 | 0,11 | 1,99 | 0,16 | 0,82 | 0,86 | 88,07 | 1,08 | 5,38 | 0,86 | 0,15 | 0,43 |
| ISC-1 | 0,11 | 0,00 | 0,13 | 2,45 | 0,13 | 1,17 | 1,05 | 85,28 | 1,31 | 6,55 | 1,05 | 0,24 | 0,52 |
| ISC-2 | 0,08 | 0,00 | 0,10 | 2,95 | 0,16 | 0,79 | 0,83 | 87,37 | 1,04 | 5,19 | 0,83 | 0,23 | 0,42 |
| ISC-3 | 0,10 | 0,00 | 0,14 | 1,58 | 0,13 | 0,96 | 0,98 | 75,99 | 0,18 | 17,84 | 0,99 | 0,24 | 0,88 |
| ISC-4 | 0,10 | 0,01 | 0,10 | 3,29 | 0,16 | 1,59 | 0,84 | 86,09 | 1,04 | 5,22 | 0,84 | 0,31 | 0,42 |
| ISC-5 | 0,13 | 0,00 | 0,10 | 2,92 | 0,14 | 1,04 | 0,77 | 87,87 | 0,96 | 4,81 | 0,77 | 0,10 | 0,38 |
| ISC-6 | 0,11 | 0,00 | 0,10 | 3,39 | 0,15 | 1,28 | 0,82 | 86,57 | 1,02 | 5,10 | 0,82 | 0,24 | 0,41 |
| ISC-7 | 0,08 | 0,00 | 0,11 | 2,94 | 0,16 | 1,04 | 0,85 | 86,92 | 1,06 | 5,31 | 0,85 | 0,25 | 0,42 |
| ISC-8 | 0,08 | 0,00 | 0,10 | 2,85 | 0,15 | 1,26 | 0,82 | 87,18 | 1,02 | 5,12 | 0,82 | 0,18 | 0,41 |
| ISC-9 | 0,09 | 0,00 | 0,11 | 2,45 | 0,16 | 1,04 | 0,86 | 87,38 | 1,07 | 5,35 | 0,86 | 0,22 | 0,43 |
| ISC-10 | 0,09 | 0,00 | 0,10 | 3,06 | 0,16 | 1,12 | 0,83 | 86,86 | 1,04 | 5,20 | 0,83 | 0,27 | 0,42 |
| ISC-11 | 0,09 | 0,00 | 0,12 | 2,39 | 0,14 | 1,21 | 0,91 | 81,22 | 0,62 | 11,47 | 0,91 | 0,28 | 0,65 |
| ISC-12 | 0,10 | 0,00 | 0,11 | 2,97 | 0,16 | 1,48 | 0,85 | 86,43 | 1,07 | 5,34 | 0,85 | 0,21 | 0,43 |
| ISC-13 | 0,08 | 0,00 | 0,11 | 2,10 | 0,16 | 0,96 | 0,88 | 87,69 | 1,10 | 5,49 | 0,88 | 0,11 | 0,44 |
| ISC-14 | 0,11 | 0,00 | 0,10 | 3,09 | 0,16 | 1,16 | 0,83 | 86,91 | 1,04 | 5,21 | 0,83 | 0,14 | 0,42 |
| ISC-15 | 0,10 | 0,00 | 0,10 | 2,00 | 0,16 | 0,64 | 0,83 | 88,54 | 1,04 | 5,22 | 0,83 | 0,11 | 0,42 |
| ISC-16 | 0,08 | 0,00 | 0,10 | 2,53 | 0,16 | 1,15 | 0,84 | 87,51 | 1,05 | 5,23 | 0,84 | 0,10 | 0,42 |
| ISC-17 | 0,08 | 0,00 | 0,11 | 2,11 | 0,16 | 0,91 | 0,88 | 87,75 | 1,09 | 5,47 | 0,88 | 0,11 | 0,44 |
| ISC-18 | 0,09 | 0,00 | 0,11 | 2,74 | 0,16 | 1,00 | 0,85 | 87,22 | 1,06 | 5,32 | 0,85 | 0,17 | 0,43 |
| ISC-19 | 0,09 | 0,00 | 0,12 | 2,05 | 0,15 | 1,09 | 0,93 | 81,45 | 0,63 | 11,73 | 0,93 | 0,17 | 0,66 |
| ISC-20 | 0,09 | 0,00 | 0,11 | 2,56 | 0,16 | 1,22 | 0,86 | 87,05 | 1,07 | 5,37 | 0,86 | 0,22 | 0,43 |
| ISC-21 | 0,12 | 0,00 | 0,10 | 3,50 | 0,15 | 1,40 | 0,82 | 86,31 | 1,02 | 5,11 | 0,82 | 0,24 | 0,41 |
| ISC-22 | 0,09 | 0,00 | 0,11 | 3,06 | 0,16 | 1,17 | 0,85 | 86,66 | 1,06 | 5,32 | 0,85 | 0,25 | 0,43 |
| ISC-23 | 0,09 | 0,00 | 0,10 | 2,96 | 0,15 | 1,39 | 0,82 | 86,92 | 1,03 | 5,13 | 0,82 | 0,17 | 0,41 |
| ISC-24 | 0,09 | 0,00 | 0,11 | 2,57 | 0,16 | 1,17 | 0,86 | 87,11 | 1,07 | 5,36 | 0,86 | 0,21 | 0,43 |
| ISC-25 | 0,09 | 0,00 | 0,11 | 2,00 | 0,16 | 0,86 | 0,87 | 87,96 | 1,09 | 5,45 | 0,87 | 0,11 | 0,44 |
| ISC-26 | 0,10 | 0,00 | 0,10 | 3,18 | 0,16 | 1,25 | 0,83 | 86,60 | 1,04 | 5,21 | 0,83 | 0,27 | 0,42 |
| ISC-27 | 0,10 | 0,00 | 0,12 | 2,50 | 0,14 | 1,34 | 0,91 | 80,94 | 0,62 | 11,49 | 0,91 | 0,28 | 0,65 |
| ISC-28 | 0,10 | 0,00 | 0,10 | 2,68 | 0,16 | 0,91 | 0,84 | 87,51 | 1,05 | 5,24 | 0,84 | 0,15 | 0,42 |
| ISC-29 | 0,08 | 0,00 | 0,11 | 2,19 | 0,16 | 0,65 | 0,87 | 87,91 | 1,09 | 5,46 | 0,87 | 0,16 | 0,44 |
| ISC-30 | 0,08 | 0,00 | 0,11 | 2,12 | 0,16 | 0,90 | 0,84 | 88,12 | 1,05 | 5,26 | 0,84 | 0,11 | 0,42 |
| IAC-1 | 0,10 | 0,01 | 0,13 | 2,01 | 0,13 | 1,39 | 1,05 | 85,51 | 1,31 | 6,56 | 1,05 | 0,21 | 0,53 |
| IAC-2 | 0,09 | 0,00 | 0,11 | 2,05 | 0,16 | 0,88 | 0,88 | 87,79 | 1,10 | 5,49 | 0,88 | 0,13 | 0,44 |
| IAC-3 | 0,08 | 0,00 | 0,11 | 1,81 | 0,17 | 0,86 | 0,89 | 87,91 | 1,11 | 5,56 | 0,89 | 0,16 | 0,45 |
| IAC-4 | 0,08 | 0,00 | 0,11 | 1,74 | 0,16 | 0,82 | 0,87 | 88,23 | 1,09 | 5,45 | 0,87 | 0,15 | 0,44 |
| IAC-5 | 0,09 | 0,00 | 0,10 | 3,35 | 0,15 | 0,62 | 0,78 | 87,70 | 0,97 | 4,87 | 0,78 | 0,21 | 0,39 |
| IAC-6 | 0,09 | 0,00 | 0,11 | 2,51 | 0,17 | 0,91 | 0,88 | 87,23 | 1,10 | 5,52 | 0,88 | 0,16 | 0,44 |
| IAC-7 | 0,09 | 0,00 | 0,11 | 1,83 | 0,16 | 0,67 | 0,86 | 88,40 | 1,08 | 5,39 | 0,86 | 0,11 | 0,43 |
| IAC-8 | 0,09 | 0,00 | 0,10 | 2,33 | 0,14 | 0,73 | 0,88 | 87,01 | 0,86 | 6,37 | 0,76 | 0,20 | 0,53 |
| IAC-9 | 0,09 | 0,00 | 0,10 | 3,00 | 0,15 | 0,98 | 0,82 | 87,26 | 1,03 | 5,15 | 0,82 | 0,17 | 0,41 |
| IAC-10 | 0,09 | 0,00 | 0,12 | 2,34 | 0,14 | 0,76 | 0,94 | 85,78 | 0,96 | 7,20 | 0,83 | 0,22 | 0,61 |
| IAC-11 | 0,10 | 0,00 | 0,10 | 2,96 | 0,15 | 0,75 | 0,79 | 87,83 | 0,99 | 4,95 | 0,79 | 0,19 | 0,40 |
| IAC-12 | 0,09 | 0,00 | 0,10 | 2,17 | 0,14 | 0,84 | 0,87 | 87,12 | 0,85 | 6,34 | 0,76 | 0,19 | 0,53 |
| IAC-13 | 0,09 | 0,00 | 0,10 | 2,84 | 0,15 | 1,08 | 0,82 | 87,36 | 1,03 | 5,13 | 0,82 | 0,16 | 0,41 |
| IAC-14 | 0,10 | 0,00 | 0,12 | 2,18 | 0,14 | 0,87 | 0,94 | 85,89 | 0,96 | 7,17 | 0,83 | 0,20 | 0,61 |
| IAC-15 | 0,10 | 0,00 | 0,10 | 2,80 | 0,15 | 0,85 | 0,79 | 87,93 | 0,99 | 4,93 | 0,79 | 0,17 | 0,39 |
| IAC-16 | 0,09 | 0,00 | 0,10 | 2,58 | 0,13 | 0,65 | 0,84 | 87,23 | 0,82 | 6,12 | 0,73 | 0,19 | 0,51 |
| IAC-17 | 0,09 | 0,00 | 0,11 | 1,92 | 0,12 | 0,43 | 0,96 | 85,80 | 0,75 | 8,14 | 0,74 | 0,24 | 0,71 |
| IAC-18 | 0,10 | 0,00 | 0,10 | 2,55 | 0,13 | 0,44 | 0,81 | 87,79 | 0,79 | 5,88 | 0,70 | 0,21 | 0,49 |
| IAC-19 | 0,08 | 0,00 | 0,10 | 2,32 | 0,13 | 0,66 | 0,87 | 87,24 | 0,84 | 6,29 | 0,75 | 0,20 | 0,53 |
| IAC-20 | 0,08 | 0,00 | 0,10 | 2,22 | 0,16 | 0,67 | 0,83 | 88,30 | 1,04 | 5,19 | 0,83 | 0,15 | 0,42 |
| IAC-21 | 0,07 | 0,00 | 0,15 | 1,82 | 0,15 | 0,42 | 0,80 | 86,61 | 0,83 | 7,97 | 0,68 | 0,12 | 0,39 |
| IAC-22 | 0,08 | 0,00 | 0,11 | 1,76 | 0,16 | 0,67 | 0,85 | 88,62 | 1,06 | 5,31 | 0,85 | 0,11 | 0,43 |
| IAC-23 | 0,08 | 0,00 | 0,11 | 1,63 | 0,16 | 0,66 | 0,86 | 88,69 | 1,07 | 5,35 | 0,86 | 0,12 | 0,43 |
| IAC-24 | 0,08 | 0,00 | 0,11 | 1,60 | 0,16 | 0,64 | 0,85 | 88,83 | 1,06 | 5,29 | 0,85 | 0,11 | 0,42 |
| IAC-25 | 0,07 | 0,00 | 0,15 | 2,58 | 0,15 | 0,62 | 0,81 | 85,48 | 0,84 | 8,04 | 0,69 | 0,19 | 0,40 |
| IAC-26 | 0,09 | 0,00 | 0,11 | 2,53 | 0,16 | 0,88 | 0,86 | 87,48 | 1,07 | 5,36 | 0,86 | 0,18 | 0,43 |
| IAC-27 | 0,08 | 0,00 | 0,11 | 2,41 | 0,16 | 0,87 | 0,86 | 87,54 | 1,08 | 5,40 | 0,86 | 0,19 | 0,43 |
| IAC-28 | 0,07 | 0,00 | 0,10 | 3,12 | 0,15 | 0,86 | 0,80 | 87,58 | 1,00 | 4,98 | 0,80 | 0,15 | 0,40 |
| IAC-29 | 0,09 | 0,00 | 0,10 | 2,96 | 0,16 | 0,76 | 0,83 | 87,48 | 1,04 | 5,18 | 0,83 | 0,18 | 0,41 |
| IAC-30 | 0,09 | 0,00 | 0,10 | 2,63 | 0,15 | 0,64 | 0,82 | 88,04 | 1,02 | 5,12 | 0,82 | 0,15 | 0,41 |
| IAC-31 | 0,09 | 0,00 | 0,11 | 2,17 | 0,16 | 0,79 | 0,87 | 87,83 | 1,09 | 5,46 | 0,87 | 0,12 | 0,44 |
| IAC-32 | 0,08 | 0,00 | 0,11 | 2,13 | 0,16 | 0,89 | 0,86 | 87,89 | 1,07 | 5,36 | 0,86 | 0,17 | 0,43 |
| IAC-33 | 0,08 | 0,00 | 0,11 | 1,72 | 0,16 | 0,95 | 0,88 | 88,07 | 1,10 | 5,48 | 0,88 | 0,14 | 0,44 |
| IAC-34 | 0,08 | 0,00 | 0,11 | 1,81 | 0,17 | 0,82 | 0,88 | 88,07 | 1,10 | 5,50 | 0,88 | 0,14 | 0,44 |
