# Supplementary material for: Data on some qualitative parameters of Carolea olive oils obtained in different areas of Calabria (Southern Italy)
Source: Data Brief. 2016 Aug 9;9:78–80. doi: 10.1016/j.dib.2016.08.009 (PMC5021706; doi:10.1016/j.dib.2016.08.009)
Supplement: Supplementary file 3 — Supplementary material [file mmc3.doc]

| samples | % Eritrodiol + Uvaol | mg/kg Eritrodiol + Uvaol |
| --- | --- | --- |
| SP-1 | 2,86 | 42,70 |
| SP-2 | 2,47 | 35,81 |
| SP-3 | 2,88 | 40,57 |
| SP-4 | 2,85 | 44,76 |
| SP-5 | 2,41 | 44,95 |
| SP-6 | 2,20 | 43,09 |
| SP-7 | 1,90 | 36,26 |
| SP-8 | 2,33 | 45,50 |
| SP-9 | 2,48 | 46,69 |
| SP-10 | 2,48 | 48,68 |
| SP-11 | 2,35 | 45,54 |
| SP-12 | 2,35 | 48,16 |
| SP-13 | 2,50 | 50,15 |
| SP-14 | 1,12 | 13,56 |
| SP-15 | 2,43 | 48,97 |
| SP-16 | 3,08 | 55,07 |
| SP-17 | 2,56 | 54,52 |
| SP-18 | 2,14 | 42,43 |
| SP-19 | 2,74 | 54,95 |
| SP-20 | 2,77 | 52,11 |
| SP-21 | 1,75 | 26,61 |
| SP-22 | 2,42 | 47,10 |
| SP-23 | 2,41 | 46,11 |
| SP-24 | 2,51 | 46,24 |
| VSE-1 | 2,48 | 36,09 |
| VSE-2 | 2,41 | 35,19 |
| VSE-3 | 2,35 | 41,13 |
| VSE-4 | 2,36 | 46,65 |
| VSE-5 | 2,54 | 39,48 |
| VSE-6 | 1,66 | 20,40 |
| VSE-7 | 1,63 | 22,21 |
| VSE-8 | 2,27 | 46,77 |
| VSE-9 | 2,21 | 41,73 |
| VSE-10 | 2,21 | 43,87 |
| VSE-11 | 1,97 | 38,30 |
| VSE-12 | 2,31 | 41,06 |
| VSE-13 | 3,16 | 72,11 |
| VSE-14 | 2,04 | 38,24 |
| VSE-15 | 2,42 | 44,49 |
| VSE-16 | 2,49 | 45,68 |
| VSE-17 | 2,39 | 47,93 |
| VSE-18 | 1,44 | 19,81 |
| VSE-19 | 2,30 | 42,39 |
| VSE-20 | 2,11 | 37,98 |
| VSE-21 | 2,49 | 46,77 |
| VSE-22 | 2,20 | 42,00 |
| VSE-23 | 2,77 | 48,48 |
| VSE-24 | 2,71 | 50,02 |
| VSE-25 | 2,40 | 43,75 |
| VSE-26 | 2,02 | 29,26 |
| VSE-27 | 2,00 | 32,40 |
| VSE-28 | 2,24 | 44,11 |
| VSE-29 | 2,24 | 45,24 |
| VSE-30 | 2,09 | 40,09 |
| VSE-31 | 2,14 | 39,80 |
| VSE-32 | 2,62 | 54,00 |
| VSE-33 | 2,22 | 42,04 |
| VSE-34 | 2,13 | 36,98 |
| VSE-35 | 1,77 | 27,48 |
| VSE-36 | 2,09 | 32,95 |
| VSE-37 | 1,92 | 29,91 |
| VSE-38 | 2,21 | 40,23 |
| VSE-39 | 1,77 | 30,61 |
| VSE-40 | 2,35 | 43,01 |
| VSE-41 | 2,43 | 44,19 |
| VSE-42 | 2,26 | 40,88 |
| VSE-43 | 2,26 | 42,92 |
| VSE-44 | 1,94 | 37,74 |
| TSA-1 | 2,56 | 33,34 |
| TSA-2 | 2,45 | 31,74 |
| TSA-3 | 2,68 | 36,23 |
| TSA-4 | 2,74 | 37,69 |
| TSA-5 | 2,85 | 39,69 |
| TSA-6 | 1,75 | 23,30 |
| TSA-7 | 1,86 | 26,40 |
| TSA-8 | 3,30 | 70,20 |
| TSA-9 | 5,33 | 129,91 |
| TSA-10 | 2,56 | 44,72 |
| TSA-11 | 3,04 | 53,16 |
| TSA-12 | 2,32 | 35,46 |
| TSA-13 | 2,34 | 39,59 |
| TSA-14 | 2,65 | 50,10 |
| TSA-15 | 2,52 | 46,34 |
| ISC-1 | 3,09 | 45,26 |
| ISC-2 | 2,16 | 40,25 |
| ISC-3 | 1,63 | 21,16 |
| ISC-4 | 2,60 | 57,09 |
| ISC-5 | 3,73 | 63,00 |
| ISC-6 | 2,85 | 54,16 |
| ISC-7 | 2,33 | 44,74 |
| ISC-8 | 2,64 | 52,33 |
| ISC-9 | 2,46 | 47,62 |
| ISC-10 | 2,49 | 47,65 |
| ISC-11 | 2,24 | 35,26 |
| ISC-12 | 2,41 | 47,57 |
| ISC-13 | 2,47 | 39,89 |
| ISC-14 | 2,67 | 46,09 |
| ISC-15 | 3,37 | 65,43 |
| ISC-16 | 2,46 | 44,04 |
| ISC-17 | 2,26 | 39,55 |
| ISC-18 | 2,30 | 39,84 |
| ISC-19 | 2,04 | 29,29 |
| ISC-20 | 2,44 | 48,09 |
| ISC-21 | 2,63 | 55,55 |
| ISC-22 | 2,10 | 45,20 |
| ISC-23 | 2,43 | 53,90 |
| ISC-24 | 2,23 | 48,50 |
| ISC-25 | 2,43 | 43,63 |
| ISC-26 | 2,27 | 48,45 |
| ISC-27 | 2,01 | 34,68 |
| ISC-28 | 2,69 | 46,59 |
| ISC-29 | 2,15 | 37,41 |
| ISC-30 | 2,48 | 44,52 |
| IAC-1 | 2,93 | 45,70 |
| IAC-2 | 2,59 | 44,71 |
| IAC-3 | 2,52 | 45,21 |
| IAC-4 | 2,48 | 43,50 |
| IAC-5 | 2,88 | 43,54 |
| IAC-6 | 2,22 | 41,82 |
| IAC-7 | 2,68 | 44,96 |
| IAC-8 | 1,76 | 28,97 |
| IAC-9 | 2,25 | 42,85 |
| IAC-10 | 2,65 | 42,64 |
| IAC-11 | 2,40 | 46,92 |
| IAC-12 | 1,78 | 30,39 |
| IAC-13 | 2,27 | 44,90 |
| IAC-14 | 2,67 | 44,48 |
| IAC-15 | 2,42 | 49,06 |
| IAC-16 | 1,50 | 26,26 |
| IAC-17 | 1,87 | 27,97 |
| IAC-18 | 1,68 | 30,24 |
| IAC-19 | 1,56 | 27,01 |
| IAC-20 | 2,23 | 40,86 |
| IAC-21 | 1,80 | 33,34 |
| IAC-22 | 2,44 | 43,22 |
| IAC-23 | 2,41 | 43,48 |
| IAC-24 | 2,39 | 42,65 |
| IAC-25 | 1,72 | 32,16 |
| IAC-26 | 2,37 | 42,28 |
| IAC-27 | 2,34 | 42,49 |
| IAC-28 | 2,15 | 48,12 |
| IAC-29 | 2,57 | 42,83 |
| IAC-30 | 2,78 | 44,25 |
| IAC-31 | 2,45 | 43,43 |
| IAC-32 | 2,35 | 44,42 |
| IAC-33 | 2,63 | 47,58 |
| IAC-34 | 2,48 | 45,26 |
