# Supplementary material for: Data on some qualitative parameters of Carolea olive oils obtained in different areas of Calabria (Southern Italy)
Source: Data Brief. 2016 Aug 9;9:78–80. doi: 10.1016/j.dib.2016.08.009 (PMC5021706; doi:10.1016/j.dib.2016.08.009)
Supplement: Supplementary file 4 — Supplementary material [file mmc4.doc]

| samples | Ethyl Palmitate | Ethyl Oleate |
| --- | --- | --- |
| SP-1 | 0,34 | 1,61 |
| SP-2 | 2,17 | 8,06 |
| SP-3 | 0,95 | 4,86 |
| SP-4 | 0,77 | 1,65 |
| SP-5 | 1,41 | 2,31 |
| SP-6 | 2,35 | 0,58 |
| SP-7 | 3,48 | 4,65 |
| SP-8 | 2,56 | 11,30 |
| SP-9 | 1,44 | 4,10 |
| SP-10 | 1,94 | 5,38 |
| SP-11 | 1,38 | 0,74 |
| SP-12 | 8,55 | 27,35 |
| SP-13 | 1,47 | 2,70 |
| SP-14 | 1,02 | 1,77 |
| SP-15 | 1,89 | 2,82 |
| SP-16 | 4,55 | 14,75 |
| SP-17 | 2,14 | 5,98 |
| SP-18 | 3,38 | 7,49 |
| SP-19 | 3,86 | 10,90 |
| SP-20 | 3,12 | 8,32 |
| SP-21 | 4,23 | 12,69 |
| SP-22 | 1,66 | 3,05 |
| SP-23 | 1,98 | 7,55 |
| SP-24 | 2,14 | 4,74 |
| VSE-1 | 1,06 | 3,31 |
| VSE-2 | 0,42 | 5,01 |
| VSE-3 | 4,84 | 12,96 |
| VSE-4 | 2,29 | 2,73 |
| VSE-5 | 6,07 | 19,13 |
| VSE-6 | 2,77 | 1,91 |
| VSE-7 | 2,53 | 6,27 |
| VSE-8 | 2,92 | 3,53 |
| VSE-9 | 5,90 | 8,75 |
| VSE-10 | 8,07 | 17,05 |
| VSE-11 | 2,98 | 1,73 |
| VSE-12 | 1,13 | 1,18 |
| VSE-13 | 2,46 | 6,26 |
| VSE-14 | 3,06 | 7,20 |
| VSE-15 | 0,85 | 0,59 |
| VSE-16 | 3,21 | 3,01 |
| VSE-17 | 2,09 | 3,13 |
| VSE-18 | 1,79 | 3,29 |
| VSE-19 | 3,03 | 7,29 |
| VSE-20 | 1,55 | 1,95 |
| VSE-21 | 1,59 | 2,88 |
| VSE-22 | 0,44 | 1,79 |
| VSE-23 | 1,05 | 1,88 |
| VSE-24 | 1,33 | 2,39 |
| VSE-25 | 0,96 | 2,41 |
| VSE-26 | 3,70 | 6,86 |
| VSE-27 | 2,41 | 4,47 |
| VSE-28 | 4,26 | 5,87 |
| VSE-29 | 5,18 | 9,47 |
| VSE-30 | 4,28 | 4,84 |
| VSE-31 | 2,05 | 1,45 |
| VSE-32 | 2,78 | 6,76 |
| VSE-33 | 3,09 | 2,37 |
| VSE-34 | 26,79 | 64,15 |
| VSE-35 | 1,67 | 2,63 |
| VSE-36 | 1,57 | 2,85 |
| VSE-37 | 1,38 | 2,86 |
| VSE-38 | 2,25 | 4,48 |
| VSE-39 | 2,17 | 4,43 |
| VSE-40 | 1,94 | 4,73 |
| VSE-41 | 1,44 | 2,17 |
| VSE-42 | 1,26 | 2,18 |
| VSE-43 | 1,82 | 2,54 |
| VSE-44 | 1,61 | 1,52 |
| TSA-1 | 0,19 | 1,02 |
| TSA-2 | 0,10 | 0,90 |
| TSA-3 | 0,51 | 2,33 |
| TSA-4 | 0,18 | 0,58 |
| TSA-5 | 0,13 | 1,31 |
| TSA-6 | 1,88 | 1,26 |
| TSA-7 | 0,83 | 1,22 |
| TSA-8 | 1,99 | 0,42 |
| TSA-9 | 0,78 | 0,56 |
| TSA-10 | 1,49 | 1,03 |
| TSA-11 | 1,82 | 0,91 |
| TSA-12 | 1,01 | 0,84 |
| TSA-13 | 0,85 | 0,84 |
| TSA-14 | 2,68 | 8,32 |
| TSA-15 | 0,39 | 0,99 |
| ISC-1 | 0,29 | 1,42 |
| ISC-2 | 2,84 | 1,85 |
| ISC-3 | 3,51 | 3,86 |
| ISC-4 | 2,41 | 3,02 |
| ISC-5 | 2,39 | 3,28 |
| ISC-6 | 2,02 | 2,01 |
| ISC-7 | 12,92 | 31,84 |
| ISC-8 | 1,40 | 1,26 |
| ISC-9 | 1,68 | 1,69 |
| ISC-10 | 2,23 | 1,90 |
| ISC-11 | 2,55 | 2,92 |
| ISC-12 | 7,84 | 14,37 |
| ISC-13 | 11,10 | 19,28 |
| ISC-14 | 7,33 | 13,07 |
| ISC-15 | 2,61 | 5,45 |
| ISC-16 | 6,10 | 11,19 |
| ISC-17 | 6,25 | 11,30 |
| ISC-18 | 7,27 | 12,24 |
| ISC-19 | 7,81 | 13,81 |
| ISC-20 | 5,28 | 6,78 |
| ISC-21 | 2,89 | 3,08 |
| ISC-22 | 14,33 | 33,88 |
| ISC-23 | 2,20 | 2,25 |
| ISC-24 | 2,46 | 2,65 |
| ISC-25 | 2,52 | 2,87 |
| ISC-26 | 3,06 | 2,92 |
| ISC-27 | 3,40 | 3,98 |
| ISC-28 | 4,85 | 5,67 |
| ISC-29 | 19,16 | 42,65 |
| ISC-30 | 3,95 | 4,56 |
| IAC-1 | 0,31 | 1,28 |
| IAC-2 | 1,03 | 5,60 |
| IAC-3 | 0,83 | 2,49 |
| IAC-4 | 1,04 | 3,48 |
| IAC-5 | 19,90 | 55,11 |
| IAC-6 | 4,26 | 15,20 |
| IAC-7 | 2,65 | 5,57 |
| IAC-8 | 0,75 | 0,52 |
| IAC-9 | 0,94 | 0,67 |
| IAC-10 | 0,72 | 0,67 |
| IAC-11 | 1,61 | 0,65 |
| IAC-12 | 2,39 | 5,10 |
| IAC-13 | 2,61 | 5,25 |
| IAC-14 | 2,34 | 5,23 |
| IAC-15 | 3,32 | 5,11 |
| IAC-16 | 0,67 | 0,72 |
| IAC-17 | 0,43 | 0,72 |
| IAC-18 | 1,36 | 0,70 |
| IAC-19 | 4,23 | 8,19 |
| IAC-20 | 1,26 | 1,94 |
| IAC-21 | 1,76 | 5,86 |
| IAC-22 | 0,62 | 2,77 |
| IAC-23 | 0,53 | 1,38 |
| IAC-24 | 0,62 | 1,81 |
| IAC-25 | 2,91 | 7,92 |
| IAC-26 | 1,46 | 4,25 |
| IAC-27 | 1,35 | 2,70 |
| IAC-28 | 4,59 | 9,77 |
| IAC-29 | 10,99 | 32,36 |
| IAC-30 | 9,13 | 24,17 |
| IAC-31 | 3,37 | 9,84 |
| IAC-32 | 1,02 | 2,34 |
| IAC-33 | 1,31 | 2,37 |
| IAC-34 | 0,75 | 1,84 |
